# Cryptic Metabolites from Marine-Derived Microorganisms Using OSMAC and Epigenetic Approaches

**DOI:** 10.3390/md20020084

**Published:** 2022-01-18

**Authors:** Cristina Pinedo-Rivilla, Josefina Aleu, Rosa Durán-Patrón

**Affiliations:** 1 Departamento de Química Orgánica, Facultad de Ciencias, Universidad de Cádiz, Puerto Real, 11510 Cádiz, Spain; cristina.pinedo@uca.es; 2 Instituto de Investigación en Biomoléculas (INBIO), Universidad de Cádiz, Puerto Real, 11510 Cádiz, Spain; 3 Instituto de Investigación Vitivinícola y Agroalimentaria (IVAGRO), Universidad de Cádiz, Puerto Real, 11510 Cádiz, Spain

**Keywords:** cryptic metabolite, marine-derived microorganism, OSMAC strategy, chemical elicitor, enzyme inhibitor, epigenetic modifier

## Abstract

Marine microorganisms have proven to be a source of new natural products with a wide spectrum of biological activities relevant in different industrial sectors. The ever-increasing number of sequenced microbial genomes has highlighted a discrepancy between the number of gene clusters potentially encoding the production of natural products and the actual number of chemically characterized metabolites for a given microorganism. Homologous and heterologous expression of these biosynthetic genes, which are often silent under experimental laboratory culture conditions, may lead to the discovery of new cryptic natural products of medical and biotechnological interest. Several new genetic and cultivation-based strategies have been developed to meet this challenge. The OSMAC approach (one strain—many compounds), based on modification of growth conditions, has proven to be a powerful strategy for the discovery of new cryptic natural products. As a direct extension of this approach, the addition of chemical elicitors or epigenetic modifiers have also been used to activate silent genes. This review looks at the structures and biological activities of new cryptic metabolites from marine-derived microorganisms obtained using the OSMAC approach, the addition of chemical elicitors, and enzymatic inhibitors and epigenetic modifiers. It covers works published up to June 2021.

## 1. Introduction

The oceans cover more than 70% of the earth’s surface and contain over 95% of the biosphere, offering a hitherto largely untapped treasure of chemical biodiversity. Moreover, marine environments include very diverse habitats with physical and chemical features that differ considerably from land-based ecosystems, resulting in marine organisms displaying a variety of structurally unique and biologically active natural products (NPs) [1,2].

In recent years, a significant number of novel metabolites with pharmacological potential have been discovered from marine microorganisms [3]. NPs discovery programmes from microorganisms mainly focus on the extraction and isolation of highly active compounds from fermentation broth and mycelium. However, these processes are becoming inefficient as they frequently lead to the rediscovery of known compounds. This has prompted researchers to develop suitable strategies to find new NPs [4].

The advent of high throughput DNA sequencing technologies has made it possible to sequence many microbial genomes over the last few years which, in turn, has helped reinvigorate interest in microbial NPs for drug discovery. In general, NPs are produced by enzymes encoded by co-localized genes in biosynthetic gene clusters. Genome sequencing has highlighted a discrepancy between the number of gene clusters potentially encoding the production of NPs and the actual total number of chemically characterized metabolites for a given organism [5]. Therefore, it is believed that a large portion of microbial gene clusters are silenced (not expressed) under standard laboratory conditions. These gene clusters are described as ‘silent’, ‘orphan’ and ‘cryptic’, and harbour an enormous reservoir of novel bioactive constituents for drug discovery [6,7].

Cryptic metabolic pathways can be accessed in the laboratory using molecular techniques [8,9,10] or cultivation-based approaches [11,12,13,14]. The number of researchers applying cultivation-dependent approaches has been increasing since this method was formalized two decades ago. The general framework was originally conceptualized by Bode et al. [15], who postulated the “One Strain—Many Compounds” (OSMAC) approach. According to this approach, each microbial strain has the potential to produce multiple compounds, but only subsets of these compounds are produced under specific growth conditions. Therefore, variations in cultivation parameters such as media composition, pH, temperature, salinity, aeration, and even the shape of vessel can induce the production of new NPs. Using this random approach that mimics natural environmental changes, Bode et al. isolated more than 100 compounds with more than 25 different skeletons from only six microorganisms [15].

Cultivation-dependent approaches can be categorized as biotic or abiotic. An abiotic approach can entail either physical or chemical changes, while a biotic approach uses organisms to stimulate NP synthesis. The most commonly used technique to elicit the production of cryptic compounds by organisms is the co-cultivation of different bacterial and/or fungal strains [16,17,18]. Recently, it has been shown that the addition of bacteriophages can also lead to the production of new NPs [19].

**Figure 1:**
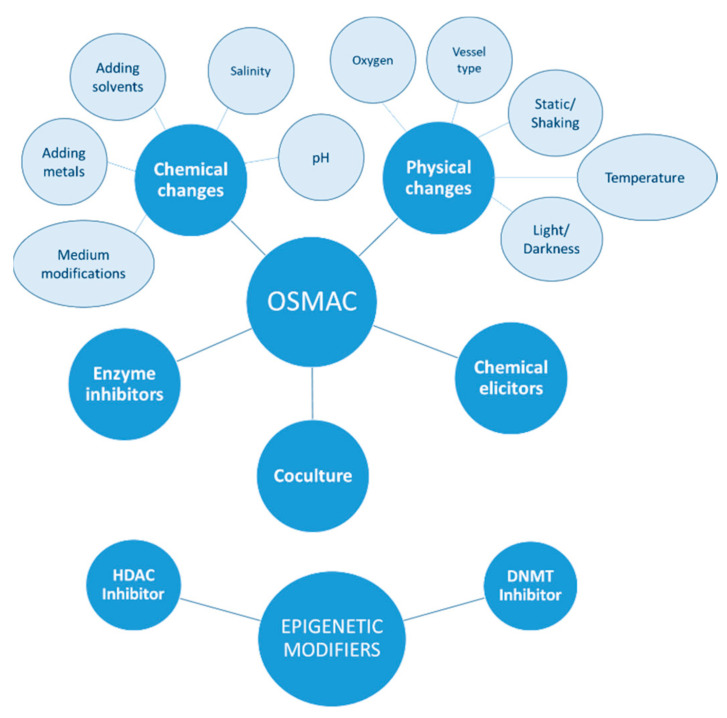
Strategies for the discovery of cryptic metabolites.

Chemical conditions within the culture system can be altered by varying the nutrient composition of the media (salts, amino acids, carbon source) [20,21,22], salinity [23,24] and pH, by changing between solid and liquid medium [25,26], and by adding solvents (DMSO or ethanol) [27] or trace-elements (halogens; metal cations such as Mg^2+^, Cu^2+^, Cd^2+^, Cr^3+^, Zn^2+^, Fe^3+^, and Ni^2+^; and rare earth metal cations such as Lt^3+^, Lu^3+^, Sc^3+^, and Y^3+^) [28,29,30]. Physical changes include growth temperature, light or darkness, oxygen concentration, vessel type and static or shaking conditions [31,32,33] (Figure 1).

Other additional strategies that do not simply modify chemico-physical growth parameters have been used to activate silent genes. For example, the use of chemical elicitors such as sub inhibitory concentrations of antibiotics have been shown to regulate gene expression at a transcriptional level, affecting 5–10% of all transcripts [34]. Enzyme inhibitors can also regulate NP production. Some chemicals such as metyrapone, tricyclazole and jasplakinolide can selectively inhibit the activity of monooxygenase and hydrolase in the biosynthetic pathway and promote the progress of other metabolic pathways [35,36].

Another extension of the cultivation-dependent approach is epigenetic activation which entails adding chemicals to the culture medium that can modulate gene expression. Chromatin remodeling by DNA methyl-transferase (DNMT) and histone deacetylase (HDAC) inhibitors can activate cryptic biosynthetic gene clusters that increase the chemical diversity of NPs. 5-Azacytidine (5-AZA) is the most common DNMT inhibitor used to reactivate genes aberrantly silenced via methylation by DNMTs and restore their normal function. Suberoyl bishydroxamic acid (SBHA), suberoylanilide hydroxamic acid (SAHA), and nicotinamide are the most common HDAC chemicals used to inhibit the deacetylation of histones and facilitate gene transcription and expression in microorganisms [37,38].

This review focuses on the chemical structures and biological activities of new cryptic metabolites from marine-derived microorganisms obtained using the OSMAC approach, the addition of chemical elicitors, enzymatic inhibitors and epigenetic modifiers. It covers articles published up to June 2021.

## 2. Alkaloids

Alkaloids are present in several microorganisms and exhibit relevant biological activity. Some of them display antiviral, antibacterial, anti-inflammatory and anticancer properties. Hence, exploration of novel or/and biologically active marine alkaloids and the study of their chemical and biological properties are now promising fields of research.

### 2.1. OSMAC Approach

**Figure 2:**
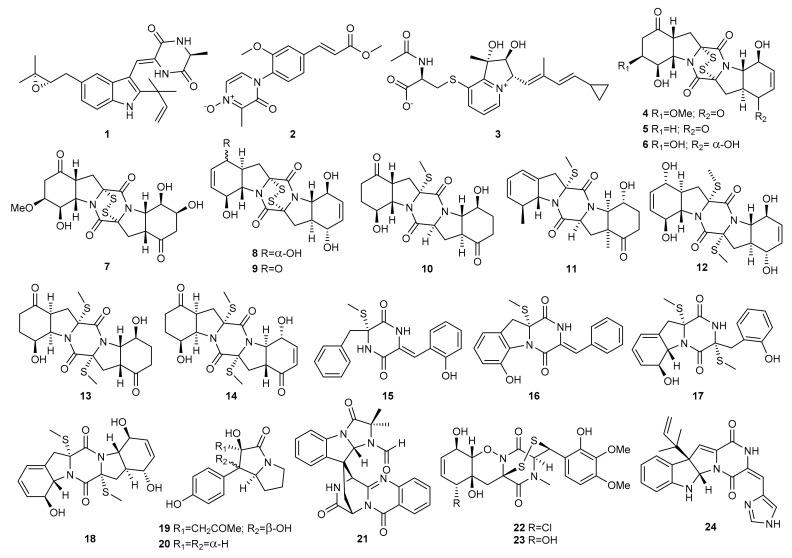
Cryptic alkaloids (**1**–**24**) from marine-derived microorganisms obtained by varying the composition of the culture medium.

It is common for gene clusters that spark the production of secondary bioactive metabolites to remain silent under experimental laboratory culture conditions. Changes in culture conditions such as nutrients, pH or temperature, have been extensively used to alter the secondary metabolic process during fungal culturing. Kamauchi et al. [39] cultured the marine derived fungus *Eurotium rubrum* (current name *Aspergillus ruber*) MPUC136 on wheat medium and Czapek-Dox-agar medium. Under rich amino acid conditions (wheat medium), it produced the new diketopiperazine compound isoechinulin D (**1**), which was not observed in Czapek-Dox-agar medium’s extracts as determined by HPLC analysis (Figure 2). The isolated compound was tested for its inhibitory activity against melanin synthesis using theophylline-stimulated B16 melanoma 4A5 cells. Compound **1** exhibited cytotoxic activity before anti-melanogenesis activity was observed.

The same strategy was used to culture the marine actinomycete *Streptomyces* sp. HZP-2216E, isolated from fresh sea lettuce *Ulva pertusa* growing on rocks in the Turtle Islet in the South China Sea. Culture of strain HZP-2216E in Gause’s liquid medium with sea salt (GMSS) resulted in the isolation of the zwitterion streptoarylpyrazinone A (**2**), a new *N*-arylpyrazinone derivative [40]. The marine actinomycete on glucose-yeast-malt (GYM) solid medium produced another zwitterion, the indolizinium alkaloid streptopertusacin A (**3**) [41]. Compound **3** exhibited moderate activity against the growth of methicillin-resistant *Staphylococcus aureus* (MRSA).

The endophytic fungus *Penicillium brocae* MA-231, derived from the marine mangrove plant *Avicennia marina*, was cultured in different media following the OSMAC approach. A series of epithiodioxopiperazines, including brocazines A–F (**4**–**9**) and penicibrocazines A–E (**10**–**14**), were obtained from the potato-dextrose broth (PDB) culture of the endophytic fungus [42,43]. Four new insaturated thiodiketopiperazine-type alkaloids, penicibrocazines F–I (**15**–**18**), together with two *p*-hydroxyphenopyrrozin alkaloids brocapyrrozins A (**19**) and B (**20**) were identified from the Czapek culture medium extract of the fungus [44].

Brocazines A–F (**4–9**) and penicibrocazines A–E (**10**–**14**) were examined for cytotoxicity against several tumour cell lines (Du145, Hela, HepG2, MCF-7, NCI-H460, SGC-7901, SW1990, SW480, and U251). Brocazines A (**4**) and B (**5**) exhibited potent activity against the SW480 tumour cell line, with IC_50_ values of 2.0 and 1.2 μM respectively, while compound **9** showed strong activity against the DU145 and NCI-H460 cell lines, with IC_50_ values of 1.7 and 0.89 μM respectively [42]. In contrast, none of the penicibrocazines (**10**–**14**) exhibited potent cytotoxic activity (IC_50_ > 10 μM) [43].

Penicibrocazines A–I (**10**–**18**) and brocapyrrozins A (**19**) and B (**20**) were also screened for antimicrobial activity against several human-, aqua-, and plant-pathogens. Penicibrocazine C (**12**) and brocapyrrozin A (**19**) exhibited inhibitory activity against *Staphylococcus aureus* (MIC = 0.25 and 0.125 μg/mL, respectively), stronger than chloromycetin used as a positive control. Penicibrocazine C (**12**) also exhibited stronger antibacterial activity than chloromycetin against *Micrococcus luteus* (MIC = 0.25 μg/mL). The antifungal activity of penicibrocazines B (**11**), D (**13**), and E (**14**) against plant pathogen *Gaeumannomyces graminis* (MIC = 0.25, 8.0, and 0.25 μg/mL, respectively) was also higher than that of the positive control amphotericin B. Brocapyrrozin A (**19**) also showed potent activity against *Fusarium oxysporum* with MIC values of 0.25 μg/mL, which was stronger than that of the positive control zeocin [43,44].

Liu et al. [25,26] observed that alteration of the fermentation media of *Penicillium adametzioides* AS-53, isolated from an unidentified sponge collected around Hainan Island in the South China Sea, affected its chemical profile. The fungus was grown in static conditions on a PDB culture medium and rice culture medium. While the solid culture medium produced acorane sesquiterpenes, the liquid culture medium led to a new spiroquinazoline derivative, *N*-formyllapatin A (**21**), along with two new bisthiodiketopiperazine derivatives, adametizines A (**22**) and B (**23**).

The biological activity of compounds **21**–**23** was evaluated. *N*-formyllapatin A (**21**) was inactive against the human-pathogen *Edwardsiella tarda* and the aqua-bacteria *Aeromonas hydrophila*, *Vibrio alginolyticus*, *V. anguillarum*, and *V. harveyi* [25]. Adametizine A (**22**) was lethal to brine shrimp (*Artemia salina*) with an LD_50_ value of 4.8 μM and had an inhibitory effect against *Staphyloccocus aureus*, *A. hydrophilia*, *Vibrio harveyi*, *V. parahaemolyticus*, and *Gaeumannomyces graminis* with MIC values of 8, 8, 32, 8, and 16 μg/mL, respectively. Adametizine B (**23**) was only active against *S. aureus* with an MIC value of 64 μg/mL [26].

Inspired by the OSMAC strategy, the fungus *Penicillium granulatum* MCCC 3A00475, isolated from a deep-sea sediment sample of the Antarctic Ocean, was investigated under different fermentation conditions. While its agitated fermentation in liquid medium produced steroids [32], static fermentation on a rice medium provided a novel roquefortine alkaloid, roquefortine J (**24**) [33]. This compound was evaluated for cytotoxic activity against HepG2 tumour cells but was found to be inactive.

**Figure 3:**
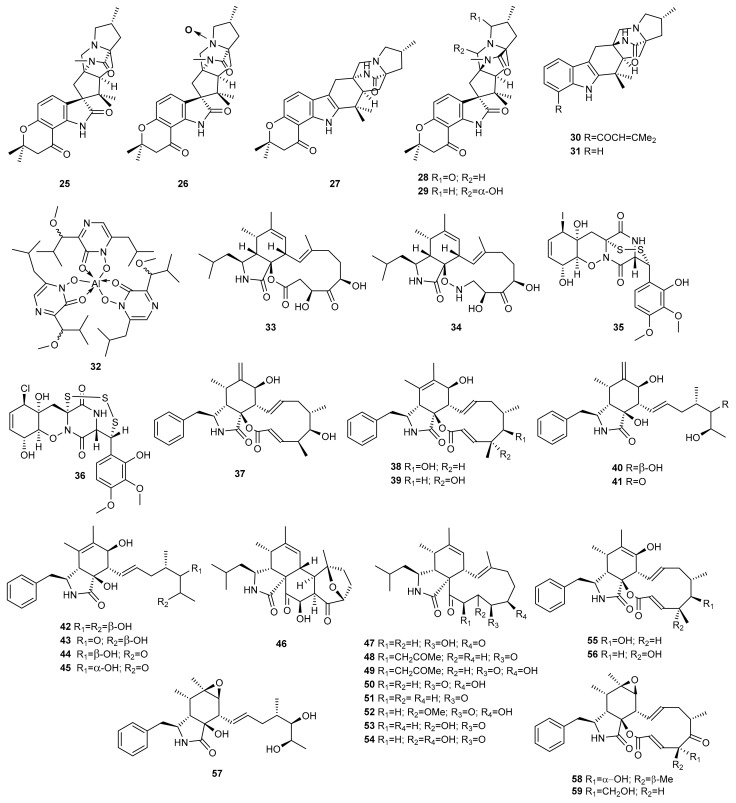
Cryptic alkaloids (**25**–**59**) from marine-derived microorganisms obtained using OSMAC approach.

Yang et al. [23,24] investigated the effect of salinity on the metabolome of the fungus *Penicillium* sp. SCSIO041218 (formerly SYFz-1), isolated from a mangrove sediment sample collected in Sanya. This SCSIO041218 strain was cultured in 0.25% and 3% NaCl rice substrate. Three new diprenylated indole alkaloids, mangrovamides A-C (**25**–**27**), were produced exclusively in the culture medium with the lower salt concentration (Figure 3). Compound **27** displayed moderate anti-acetylcholinesterase activity with an IC_50_ value of 58.0 µM. In contrast, none of the compounds (**25**–**27**) proved to be toxic against a panel of 10 human tumour cell lines (H1975, U937, K562, BGC823, MOLT-4, MCF-7, A549, Hela, HL60, and Huh-7).

Culture of the SCSIO041218 strain in 1% NaCl PDB substrate resulted in the discovery of four new prenylated indole alkaloids (**28**–**31**). These compounds were tested for anti-allergic bioactivity on IgE-mediated rat mast RBL-2H3 cells, but none were active [45].

High salt concentration is not suitable for fungus survival due to hyperosmosis, but it could activate some silenced genes. Peng et al. [46] used HPLC analysis to investigate the chemodiversity of the metabolites of the marine coral-derived *Aspergillus ochraceus* LCJ11-102 using different salts and concentrations. The LCJ11-102 strain produced similar products under NaCl, NaBr, KCl, MgCl_2_, CaCl_2_ and no salt conditions. However, it produced a new aluminium chelate, ochralate A (**32**) in a nutrient-limited medium containing 10% NaI. This compound showed antimicrobial activity against *Enterobacter aerogenes* with a MIC value of 18.9 µM.

Following a similar strategy, the wetland fungus *Aspergillus flavipes* PJ03-11 was cultured on rice medium supplemented with 1mM NaBr. Flavichalasines N (**33**) and O (**34**), two new cytochalasans absent in the bromine-untreated medium, were produced. Compound **34** possesses a particular nitrogen-oxygen heterocycle at the macrocyclic ring rather than carbocycle or oxygen heterocycle found in other cytochalasans. Both compounds (**33**, **34**) were cytotoxic against human cancer cell lines HL-60, THP1 and PC3 with IC_50_ values ranging from 3.48 to 15.10 µM [47].

The marine-derived fungus *Trichoderma* sp. TPU199 (cf. *T. brevicompactum*) isolated from a red algae in Palau is able to use halide ions from sodium halides in the medium to produce halogenated epidithiodiketopiperazine derivatives. Hence, supplementation of the freshwater medium with 3% NaI led to the isolation of a new iodinated derivative, iododithiobrevamide (**35**), which was not observed in the freshwater medium. Although Br derivatives have reportedly been produced by fermentation with inorganic bromides, it is rare to obtain an I derivative by fermentation with NaI [48].

A culture of *Trichoderma* sp TPU199 in natural seawater medium supplemented with 1% DMSO produced an unprecedented epitrithiodiketopiperazine, chlorotrithiobrevamide (**36**) with a trisulfide bond between the α-and β-positions of two amino acid residues. This compound was not produced in a freshwater medium or freshwater supplemented with DMSO. Hence, the combination of DMSO and NaCl in seawater may be a requisite for induction of the trisulfide compound [27].

The effect of **36** on the proliferation of human colon cancer HCT-15 and T-cell leukemia Jurkat cells was evaluated. Chlorotrithiobrevamide (**36**) was mildly cytotoxic against Jurkat cells with an IC_50_ value of 16 µM, and markedly reduced the activity of HCT-15 cells [27].

Gu et al. performed a chemical analysis of the marine-derived fungus *Spicaria elegans* (current name: *Mariannaea elegans*) KLA03 using the OSMAC approach. Culture of the fungus under static conditions for 25 days in liquid medium produced nine new 10-phenylcytochalasins, Z_7_-Z_15_ (**37**–**45**) [49,50]. Changes in culturing parameters of *S. elegans* induced a shift in the biosynthesis of cytochalasans. The dominant production of 10-phenylcytochalasins with a phenylalanine residue was eliminated under shaking conditions for eight days in a soybean medium, and leucine was involved in a new biosynthetic pathway leading to the production of the novel spicochalasin A (**46**) and five new aspochalasins, M-Q (**47**–**51**) [51].

A detailed analysis of the aspochalsin structures shows that their diversity results from different degree of oxidation–reduction at C17–C19. Hence, longer culture time could help to change the oxidation–reduction effect and produce more analogues. To increase the diversity of aspochalasin analogues, *S. elegans* was cultured under shaking conditions for 14 days in a soybean medium. As a result, three new analogues, aspochalasins R-T (**52**–**54**), were obtained [52].

The growth inhibition effect of these compounds against different cancer cell lines was also evaluated and cytochalasins Z_7_-Z_11_ (**37**–**41**) proved to be mildly cytotoxic against the A-549 cell line with IC_50_ values of 8.8, 21.0, 8.7, 9.6, and 4.3 µM, respectively [49,50]. Spicochalasin A (**46**) and aspochalasin M (**47**) were found to be moderately cytotoxic against human leukemic HL-60 cells, with IC_50_ values of 19.9 and 20.0 µM, respectively [51].

The oxidative reactions of cytochalasins are believed to be catalysed by P-450 mono-oxygenases. Therefore, the use of monooxygenase-dependent P-450 inhibitors could be used to obtain new derivatives. Following this strategy, *S. elegans* was treated with metyrapone, a cytochrome P-450 inhibitor. Two new 7-deoxycytochalasins, Z_7_ (**55**) and Z_9_ (**56**), were produced, which are the biosynthetic precursors of cytochalasins **38** and **39**, respectively [53]. Compounds **55** and **56** were evaluated for their cytotoxicity against the A-549 and P-388 cell lines. Compound **55** was moderately cytotoxic against the A-549 cell line with an IC_50_ value of 15.0 μM, while **56** was inactive [53].

Cytochalasins are biosynthesized by the formation of an acetate- and methionine-derived octaketide or nonaketide chain, and the attachment of an amino acid. Since the type of cytochalasin depends on the amino acid incorporated as a structural subunit, the addition of amino acids to the culture medium could lead to different types of novel cytochalasins. To test this hypothesis, *S. elegans* KLA03 was cultivated under shaking and static conditions in a liquid medium supplemented with a variety of amino acids. The addition of d-tryptophan to *S. elegans* during its cultivation under static conditions led to two new 10-phenylcytochalasins, Z_21_ (**57**) and Z_22_ (**58**), which proved to be cytotoxic against A-549 cell lines with IC_50_ values of 8.2 and 20.0 µM, respectively. Similarly, the addition of l-tryptophan afforded the new 10-phenylcytochalasin Z_23_ (**59**) [54].

**Figure 4:**
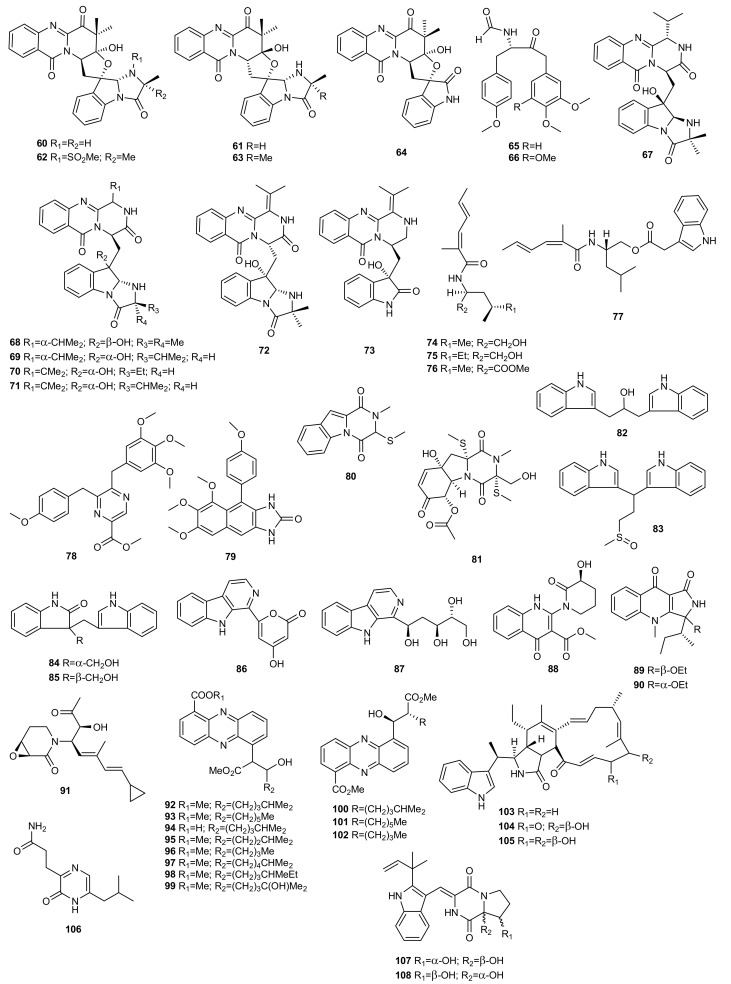
Cryptic alkaloids (**60**–**108**) from marine-derived microorganisms obtained using OSMAC and epigenetic approaches.

Following a similar strategy, the Lan group studied the impact of amino acid-supplemented media on the metabolic profile of the marine-derived fungus *Scedosporium apiospermum* (current name: *Pseudallescheria boydii*) F41-1, isolated from the inner tissue of the soft coral *Lobophytum crassum*. Culture of the fungus in glucose–peptone–yeast (GPY) medium supplemented with l-tryptophan, l-phenylalanine, l-threonine, and d,l-methionine led to the discovery of 14 new alkaloids, scedapins A-G (**60**–**66**) and scequinadolines A-G (**67**–**73**), in addition to other known alkaloids (Figure 4). Scedapins A-E (**60**–**64**) possess a rare skeleton composed of a pyrazinoquinazolinedione and an imidazoindolone/indolone linked by a tetrahydrofuran ring. Scequinadolines A (**67**) and D (**70**) displayed significant antiviral activity against the J8CC recombinant virus of hepatitis C with EC_50_ values of 128.60 and 110.35 μM, respectively. This was the first report of a fumiquinazoline derivative with anti-HCV activity [20].

Lan’s group again adopted the amino acid strategy to encourage the marine-derived fungus *Dichotomomyces cejpii* (current name: *Aspergillus cejpii*) F31-1, associated with the soft coral *Lobophytum crassum,* to generate alkaloids. The addition of l-tryptophan and l-phenylalanine to GPY medium led to the isolation of four aliphatic amides, dichotomocejs A-D (**74**–**77**), two alkaloids, dichotomocejs E (**78**) and F (**79**), and two diketopiperazines-type alkaloids, dichocerazines A (**80**) and B (**81**). Dichotomocej A (**74**) exhibited a moderate inhibitory effect against the human rhabdomyosarcoma cell line RD with an IC_50_ value of 39.1 µM. Dichotomocejs E (**78**) and F (**79**) were assayed for cytotoxic activity against a macrophage cell line (RAW264.7) but exhibited no significant inhibitory effect [55,56].

Indole alkaloids are biosynthesised by coupling of the inessential amino acid tryptophan with other amino acids and structural fragments. This prompted Lan’s group to use the same strategy to enhance the production of new indole alkaloids biosynthesised by the marine fungus *Pseudallescheria boydii* F44-1, isolated from the soft coral *Sarcophyton* sp. collected in the Sanya Coral Reef National Natural Reserve in China. They cultivated the fungus in GPY medium and GPY medium fed with l-tryptophan, l-phenylalanine, l-methionine and l-threonine. According to the HPLC profiles, the extract of the medium supplemented with amino acids produced more metabolites and two new bisindole alkaloids, pseudboindoles A (**82**) and B **(83**) were identified [57].

The cytotoxic activity of the compounds **82** and **83** was tested against eight cancer cell lines, including the human lung cancer cell lines A549 and GLC82, human nasopharyngeal carcinoma cell lines CNE1, CNE2, HONE1 and SUNE1, and human hepatoma carcinoma cell lines BEL7402 and SMMC7721. Both compounds were inactive (IC_50_ > 200 μM) [57].

Similarly, the marine derived fungus *Fusarium* sp. XBB-9 collected from Sanya, China, was cultured in GPY medium supplemented with l-tryptophan and l-phenylalanine as precursors, following the OSMAC approach, to enhance as many biosynthetic pathways as possible. As a result, a pair of novel bisindole alkaloid enantiomers, (+)- and (–)-fusaspoid A (**84**, **85**) were obtained. These compounds were assayed for cytotoxic activity against the HCT-15 and RKO cell lines, but none were active [58].

Following a similar strategy, the marine gorgonian-derived fungus *Aspergillus* sp. SCSIO 41501 (formerly A*spergillus* sp. SCSGAF 0076) was cultured in basic culture medium supplemented with l-tryptophan. An increase in the concentration of the amino acid from 0.05% to 0.2% resulted in the isolation of two new β-carboline alkaloids, aspergillspins A (**86**) and B (**87**), and three new quinolone alkaloids, aspergillspins C (**88**) and E (**90**) Compounds **86**–**88** were evaluated for their cytotoxic (HL60, HepG2 and MCF-7 cell lines) and antibacterial (*Bacillus subtilis* and *Escherichia coli*) effect, but none of them exhibited any activity [59].

Shi et al. [60] adopted the metal stress strategy to enhance the expression of antimicrobials in hydrothermal vent actinomyces. According to this technique, the actinomycete *Streptomyces* sp. WU20, isolated from metal-rich hydrothermal vents in Taiwan Kueishantao, was cultured in a nickel-rich medium. The antimicrobial activity of the nickel-treated group was more effective than the control group. Chemical analysis of the Ni^2+^ stressed culture led to a novel cyclizidine analogue (**91**) exhibiting antibiotic properties against the Gram-positive bacterium *Bacillus subtilis*, with an MIC value of around 32 µg/mL.

As a direct extension of the OSMAC approach, the addition of chemical elicitors has also been used to activate silent genes. Several studies support the idea that antibiotics at subinhibitory concentrations induce transcriptional changes in bacteria. Taking this into account, the sponge-derived marine *Streptomyces* sp. HB202 was treated with subinhibitory concentrations of tetracycline and bacitracine and exhibited an increase in and modulation of the production of new phenazines, streptophenazines A-H (**92**–**99**). Moderate amounts of streptophenazines A-D (**92**–**95**) were formed in trypticase soy broth 10 (TSB10) without antibiotics, while the addition of 1.5 µg/L of tetracycline led to a significant increase in their concentration. Tetracycline also induced the formation of significant amounts of streptophenazines F (**97**) and G (**98**), only trace amounts of which were found in the antibiotic-free medium. Moderate enhancement of the concentration of streptophenazine H (**99**) was observed after pulse application of bacitracin to TSB10 medium [61]. The total synthesis of streptophenazines A (**92**), B (**93**) and E (**96**) has allowed for the correction of their structures to **100**–**102** [62].

Phenazines **92**–**99** were tested for their antibiotic activity. Streptophenazines C (**94**) and H (**99**) showed moderate activity against *Bacillus subtilis*, both with MIC values of 15.6 µg/mL. Only streptophenazine C (**94**) was also moderately active against *Staphylococcus lentus*, with a MIC value of 46.9 µg/mL [61].

Christian et al. [36] studied the impact of potent cytoskeletal inhibitors on the marine-derived fungus *Phomopsis asparagi*, obtained from a U.S. Virgin Island collection of the sponge *Rhaphidophlus juniperinus* (sensu Duchassaing & Michelotti, 1864). Cultures of the fungus with the F-actin inhibitor jasplakinolide resulted in the production of three new chaetoglobosin analogues, chaetoglobosin-510 (**103**), -540 (**104**), and -542 (**105**). Compound **105** was cytotoxic against C38 murine colon and L1210 leukaemia cancer cell lines. It also disrupted actin microfilaments in A-10 rat aortic smooth muscle cells at 1 µg/mL.

### 2.2. Epigenetic Approach

Epigenetic activation is another strategy extensively used to unlock cryptic biosynthetic gene clusters in the microbial metabolome. Liu et al. [63] applied this method to induce the production of new metabolites from the marine algicolous fungus *Aspergillus versicolor* OUCMDZ-2738. Vorinostat (SAHA) added to the culture medium led to the isolation of a new naturally occurring diketopiperazine derivative, 3-[6-(2-methylpropyl)-2-oxo-1*H*-pyrazin-3-yl]propanamide (**106**), and (+)- and (−)-brevianamide X (**107**, **108**), which were exclusively observed in the presence of the epigenetic modifier.

All the compounds (**106**–**108**) were evaluated for antibacterial activity against pathogenic microorganisms, including bacteria (*Bacillus subtilis* ATCC6051, *Clostridium perfringens* ATCC13048, *Staphylococcus aureus* ATCC6538, *S. aureus* ATCC25923, *Pseudomonas aeruginosa* ATCC10145, and *Escherichia coli* ATCC11775), and yeasts (*Candida albicans* ATCC10231 and *C. glabrata* ATCC2001). In addition, based on data reported for these kinds of compounds, its inhibitory effect against α-glucosidase from *Saccharomyces cerevisae* was tested. However, none of the compounds tested was active [63].

## 3. Peptides

Peptides are synthetized by organisms as a part of their structural components. As primary metabolites they are crucial to the normal development of life and as secondary metabolites they provide microorganisms with environmental advantages. They possess biological activities relevant to the pharmaceutical and other industries. The number of microbial peptides found to exhibit biological activity is constantly increasing and marine microorganisms are a relevant source of compounds of this nature.

### 3.1. OSMAC Approach

**Figure 5:**
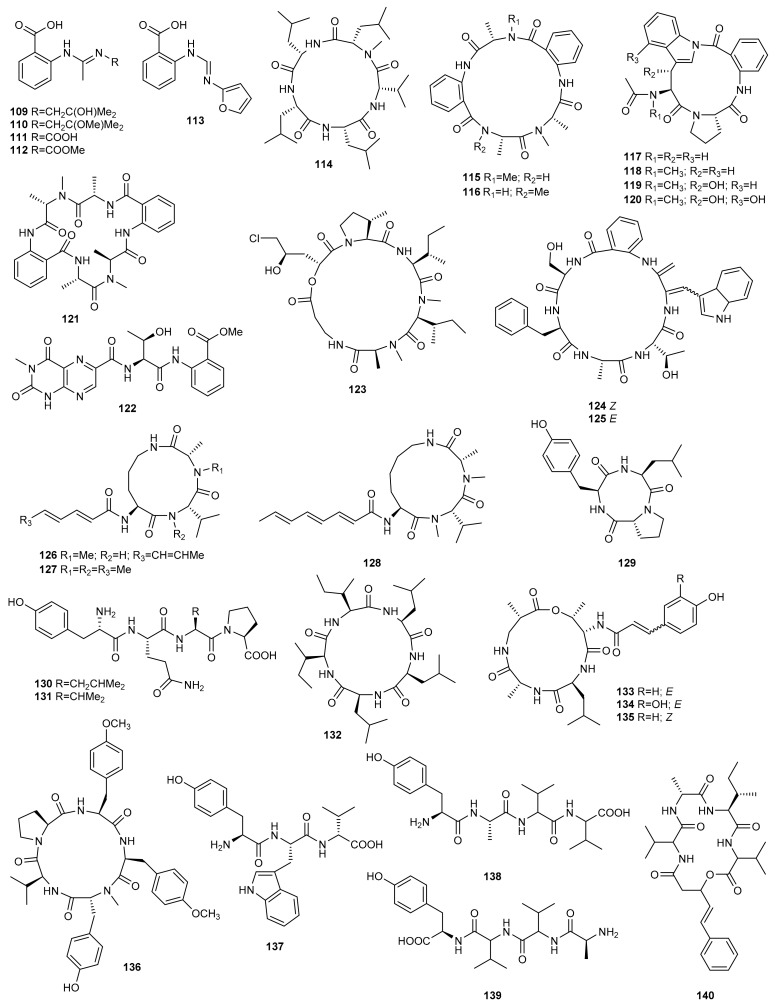
Cryptic peptides (**109**–**140**) from marine-derived microorganisms obtained using OSMAC and epigenetic approaches.

Among the various strategies developed to discover new marine peptides, changes in fermentation conditions have proven to be a promising method to awaken the silent gene clusters leading to the biosynthesis of these compounds. Thus, alterations in the cultivation parameters of the deep-sea sediment-derived fungus *Penicillium paneum* SD-44 resulted in the isolation of five new anthranilic acid derivatives, penipacids A-E (**109**–**113**) (Figure 5). These compounds were detected when the strain SD-44 was cultured in a 500 L fermentator under shaking conditions but were absent in a static culture on rice medium [64].

Penipacids A-E (**109**–**113**) were evaluated for antimicrobial and cytotoxic activity. None of them displayed antimicrobial activity against the bacteria *Staphylococcus aureus* and *Escherichia coli*, and the plant-pathogenic fungi *Alternaria brassicae*, *Fusarium graminearum* and *Rhizoctonia cerealis* (current name: *Ceratobasidium cereale*). Penipacids A (**109**) and E (**113**) exhibited cytotoxicity against human colon cancer RKO cell line with an IC_50_ value of 8.4 and 9.7 µM, respectively [64].

Gulder et al. [31] used the same strategy to stimulate the production of secondary metabolites of the marine fungus *Asteromyces cruciatus* 763, collected at La Jolla shore in San Diego (USA). They cultivated the fungus under 14 different growth conditions varying the nutrient composition of the media, incubations times, extraction procedures, UV-light exposure, and temperature. *A. cruciatus* extracts grown in a Czapek-Dox medium fed with cofactors or with the amino acids arginine, asparagine and glutamic acid as a nitrogen source instead of NaNO_3_, afforded the new pentapeptide lajollamide A (**114**). This compound exhibited weak antibacterial activity against *Bacillus subtilis* (61%) and *Staphylococcus epidermidis* (30%) at a concentration of 100 μM, and no cytotoxic or enzyme inhibition activity.

Following the OSMAC strategy, the metabolome of the marine-derived fungus *Aspergillus versicolor* ZLN-60, isolated from the sediment of the Yellow Sea, was studied using different culture media to maximize the production of new secondary metabolites. Culture of the fungus in a liquid medium led to two new anthranilic acid-containing cyclic pentapeptides, versicotides A (**115**) and B (**116**) [65]. The HPLC-UV profile changed dramatically when this original liquid culture medium was changed to a solid rice-based medium. Investigation of the extract of the solid-phase culture led to the discovery of four new cyclic peptides, psychrophilins E−H (**117**–**120**), and versicotide C (**121**). Psychrophilins are rare fungal cyclic peptides containing amide groups composed of anthranilic acid and indole motifs in the macrocycle. Biological assays revealed that psychrophilin G (**119**) exhibits potent lipid-lowering effects at a dose of 10 μM [66].

Similarly, Özkaya et al. [67] studied the metabolic profile of the sponge-associated fungus *Aspergillus carneus* using different growth media. This fungal strain was fermented on three different media including solid rice medium with and without sea salt and modified Czapek medium. Prominent variations in the chemical compositions of the extracts were found. The lumazine-containing peptide isoterrelumamide A (**122**) was only found when the fungus was cultivated on modified Czapek medium. This compound showed no antitumour or antimicrobial activity.

The OSMAC strategy was used to stimulate the production of secondary metabolites of the marine-derived fungus *Trichothecium roseum*, isolated from marine driftwood from the intertidal zone of Lingshan Island in China. Culture of this strain in PDB and rice media exhibited different metabolomic profiles. While the solid culture medium produced known destruxin cyclodepsipeptides, the liquid culture medium led to a new trichomide cyclodepsipeptide (**123**). Compound **123** exhibited significant cytotoxic activity against the human cancer cell lines MCF-7, SW480, and HL-60, with IC_50_ values of 0.079, 0.107, and 0.149 µM, respectively. It was also lethal to brine shrimp (*Artemia salina*) with an LD_50_ value of 0.48 µM, and exhibited moderate nematicidal activity against *Heterodera avenae* with an LC_50_ value of 94.9 µg/mL [68].

Zheng et al. [69] explored the effect of high-salt stress on the production of secondary metabolites from halotolerant *Aspergillus sclerotiorum* PT06-1, isolated from the Putian Sea Salt Field in China. The culture of this strain in different salt media exhibited distinct metabolite patterns based in TLC and HPLC profiles. Two novel cyclic hexapeptides containing both anthranilic acid and dehydrotryptophan residues, sclerotides A (**124**) and B (**125**), were identified from the metabolites in a nutrient-limited medium containing 10% NaCl.

Compounds **124** and **125** are photointerconvertible and could be interconverted via a radical reaction initiated by direct photoisomerization. Both compounds exhibited moderate antifungal activity against *Candida albicans* with MIC values of 7.0 and 3.5 µM, respectively. Compound **125** also showed weak cytotoxicity against the HL-60 cell line (IC_50_ = 56.1 µM) and selective antibacterial activity against *Pseudomonas aeruginosa* (MIC = 35.3 µM) [69].

Fermentation of *Aspergillus sclerotiorum* PT06-1 in a nutrient-rich medium with 10% salt concentration exhibited different TLC and HPLC profiles from those in the nutrient-limited medium. Chemical investigation resulted in the identification of three new aspochracin-type cyclic tripeptides, sclerotiotides A-C (**126**–**128**), in addition to others that were artifacts probably formed during fermentation or subsequent isolation steps. Sclerotiotides A (**126**) and B (**127**) showed selective antifungal activity against *Candida albicans* with MIC values of 7.5 and 3.8 µM, respectively, while no cytotoxicity or antibacterial activity was observed [70].

As a direct extension of the OSMAC approach, trace elements have also been used to activate silent genes. Since microorganisms have a high biosorption capacity for rare earth elements, the marine-derived fungus *Penicillium citrinum*, isolated from the marine sponge *Petrosia* sp, was cultured in malt medium supplemented with 50 μM ScCl_3_. Chemical investigation led to the isolation of three new peptide derivatives **129**–**131**, which were detected only in ScCl_3_-treated cultures. These compounds were evaluated for their cytotoxic and antibacterial activity. Compound **129** showed weak antibacterial activity against *Staphylococcus aureus* 503 and *Pseudomonas aeruginosa* 9027, while compounds **130** and **131** showed moderate cytotoxic activity against HCT-15 and MCF-7 cell lines [29].

Marine hydrothermal microorganisms respond rapidly to changes in the concentration and availability of metals in their environment, which is heavily impacted by elevated levels of heavy metals. Based on this observation, Ye et al. cultured the hydrothermal fungus *Aspergillus clavatus* C2WU, isolated from *Xenograpsus testudinatus,* in the absence and presence of ZnSO_4_. As a result, a new cyclopeptide, clavatustide C (**132**), was produced only in the culture treated with Zn in response to abiotic stress [71].

Qi et al. [21,72] adopted the amino acid–directed strategy to obtain new peptides from the fungal strain *Aspergillus* sp. SCSGAF 0076 (re-numbered as SCSIO 41501) isolated from the South China Sea gorgonian *Melitodes squamata*. As l–tryptophan is an important precursor for the synthesis of indole derivatives in microorganisms, they cultivated the fungal strain in different concentrations of this amino acid. Three new cyclic tetrapeptides, aspergillipeptides A-C (**133**–**135**) were obtained in a basic culture medium with 0.05 % of l-tryptophan [72]. An increase in the concentration of the amino acid to 0.2% and the addition of 0.5% methanol to the culture medium led to a new cyclic pentapeptide and three new linear peptides, aspergillipeptides D–G (**136**–**139**) [21].

Aspergillipeptide C (**135**) exhibited strong antifouling activity against *Bugula neritina* larvae settlement with an EC_50_ value of 11 μg/mL and LC_50_ > 300 mg/mL [72]. Aspergillipeptides D (**136**) and E (**137**) showed antiviral activity against herpes simplex virus type 1 (HSV-1) with IC_50_ values of 9.5 and 19.8 μM, respectively, at non-cytotoxic concentrations against a Vero cell line. Moreover, **136** displayed antiviral activity against acyclovir-resistant clinical isolates of HSV-1 at a concentration of 12.5 μM with about a 50% inhibition rate [21].

### 3.2. Epigenetic Approach

An epigenetic approach was used to study the secondary metabolism of the marine-derived fungus *Microascus* sp. 098059A, isolated from shallow water sediments collected in Florida. The fungus was cultured in a Czapek-Dox medium with artificial seawater in the presence of 10^−4^ M of SAHA. The RP C18 LC-ELSD-MS profile of the extract con SAHA revealed the presence of a new cyclodepsipeptide, EGM-556 (**140**), that was not present in the control culture [73].

## 4. Polyketides

Polyketides are an important family of natural compounds with a wide range of biological activity. Microorganisms produce a wide variety of compounds with this chemical skeleton and a growing number have been isolated from marine microorganisms over the last decade, making this a potential source of polyketides with new structures and interesting activity. 

### 4.1. OSMAC Approach

Abiotic strategies such as chemical stress (heavy metals) and changes in fermentation conditions (light, pH, temperature and different media), have long been known to induce notable changes and to unlock cryptic biosynthetic gene clusters in the microbial metabolome [74].

**Figure 6:**
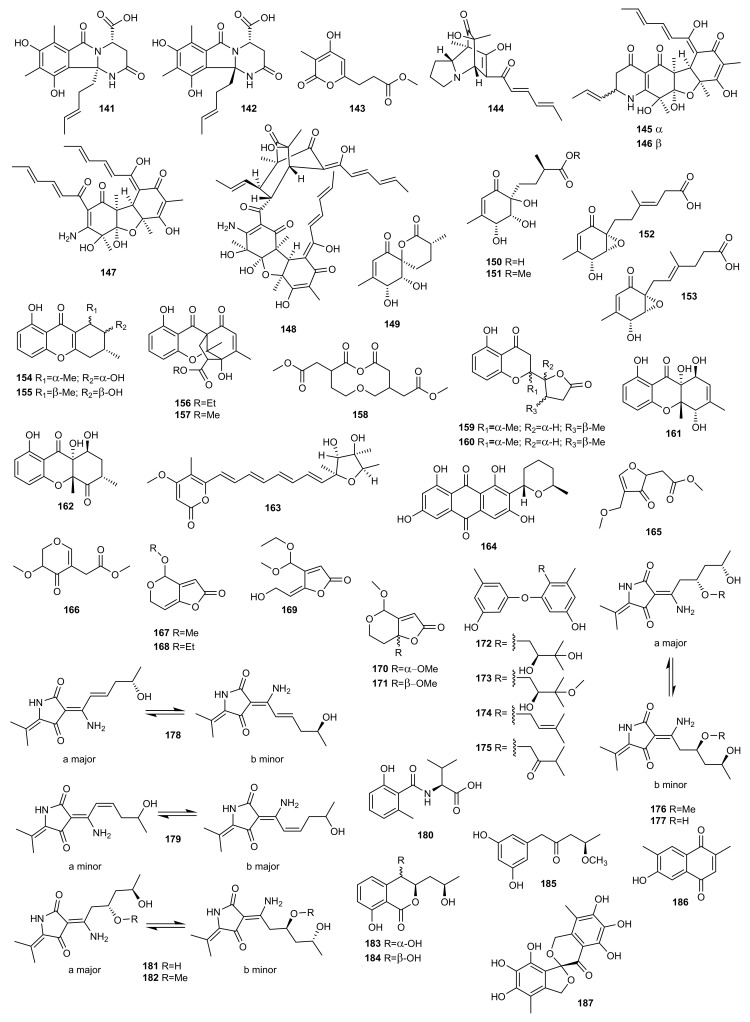
Cryptic polyketides (**141**–**187**) from marine-derived microorganisms obtained using the OSMAC approach.

Sorbicillinoids are a wide family of polyketides with very diverse carbon skeletons and biological activities. Only a few cases of nitrogen-containing sorbicillinoid analogues have been reported [75,76]. Thus, in order to find new bioactive molecules, Zhang et al. [75] isolated two new nitrogen-containing sorbicillinoids named sorbicillasins A (**141**) and B (**142**), and a new 3,4,6-trisubstituted α-pyrone derivative, scirpyrone K (**143**), from the marine-derived fungus *Phialocephala* sp. FL30r using the OSMAC strategy. A mannitol-based medium was used to culture the fungal strain, obtaining an extract whose HPLC-UV profile differed from those generated previously from culture on a potato-based medium (Figure 6).

Cytotoxicity against K562 and MGC-803 cell lines and radical scavenging activity against 1,1-diphenyl-2-picrylhydrazyl radical 2,2-diphenyl-1-(2,4,6-trinitrophenyl) hydrazyl (DPPH) of the new compounds **141**–**143** were evaluated. All were found to be non-cytotoxic. Compound **143** exhibited weak activity against DPPH with an IC_50_ value of 27.9 μM (ascorbic acid was used as a positive control with an IC_50_ value of 14.2 μM), whereas compounds **141** and **142** were not active.

Sorbicillinoids were also isolated from other fungal genera. Guo et al. used different culture conditions to maximize the ability of marine-derived fungus *Penicillium* sp. F23-2 to produce bioactive molecules [77,78]. These authors identified five new nitrogen-containing sorbicillinoids from this fungus named sorbicillamines A−E (**144**–**148**) when the culture conditions were changed from static to shaking fermentation in PYG medium containing peptone, yeast powder, and glucose [77]. 

Subsequently, the study of the metabolic profile of the deep-sea-derived fungus *Penicillium* sp. F23-2 on a rice-based solid medium indicated the presence of new compounds. The ambuic acid analogues penicyclones A−E (**149**–**153**) were isolated from the extract which exhibited antimicrobial activity against the Gram-positive bacterium *Staphylococcus aureus* [78].

Following the OSMAC approach, the fungus *Penicillium* sp. SCSIO041218 (before SYFz-1), isolated from a mangrove soil sample, was cultured on various media with different salt concentrations. Two new xanthone derivatives, penixanthones A (**154**) and B (**155**), were obtained from the 3% NaCl rice substrate [24]. In contrast, two new polycyclic chromones, penixanthones C (**156**) and D (**157**), and a new macrodiolide, mangrovlide A (**158**), were isolated from the culture on the 0.25% NaCl rice substrate [35]. Four new chromone derivatives (**159**–**162**) were also isolated from the 1% NaCl PDB substrate [45].

The cytotoxicities of compounds **154**–**162** were evaluated. Compound **154** showed weak cytotoxicity against the H1975, MCF-7, K562, and HL7702 cell lines at a concentration of 30 μM [24]. Compounds **157** and **158** exhibited weak activity against K562, MCF-7, and Huh-7 cell lines, with IC_50_ values of 55.2–67.5 µM [35]. Compounds **159**–**162** tested negative for anti-allergic bioactivity on IgE-mediated rat mast RBL-2H3 cells [45].

Taking into account the potential of the OSMAC strategy to induce the production of new metabolites, Adpressa et al. [79] conducted a comparative metabolomic study of a marine derived fungus, *Aspergillus terreus*. The study consisted of testing eleven different culture conditions and was guided by a multivariate analysis of LC/MS data from the organic extracts leading to the isolation of several secondary metabolites. The study concluded that some culture conditions produced substantial differences in the metabolic profile and bioactivity of the extracts. The compound 7-desmethylcitreoviridin (**163**), a new cytotoxic compound, was described in this study for the first time. 

Özkaya et al. [67] studied the metabolic profile of the sponge-associated fungus *Aspergillus carneus* in three media, solid rice medium containing sea salt, modified Czapek medium and solid rice medium with no sea salt. Prominent variations in the chemical compositions of the extracts were exhibited. The new polyketide 5′-epi-averufanin (**164**) was isolated from the extracts of the fermentations when the fungus was cultivated on solid rice medium with and without sea salt. Compound **164** was found to be active against the Gram-positive bacteria *Staphylococcus aureus* ATCC 700699 and *Enterococcus faecium* ATCC 35667 with MIC values of 4.6 and 9.3 μg/mL, respectively. 

The secondary metabolism of the marine-derived fungus *Aspergillus giganteus* NTU967 was studied after testing the antimicrobial activity of the extracts against *Escherichia coli*, *Staphylococcus aureus*, *Candida albicans*, and *Cryptococcus neoformans*. The ethyl acetate extract from the control fermentation exhibited significant inhibition against *S. aureus* and *C. neoformans*. This prompted the culture of *Aspergillus giganteus* NTU967 in liquid and solid medium, resulting in the isolation and identification of aspergilsmin A-G (**165**–**171),** seven previously unreported highly oxygenated polyketides [80]. The anti-tumoral activity of the compounds isolated was also tested, with compound **167** exhibiting the best activity against human hepatocellular carcinoma SK-Hep-1 cells and prostate cancer PC-3 cells. 

In the search for new bioactive metabolites from the fungus *Aspergillus versicolor* ZLN-60 derived from a marine sponge *Petrosia* sp. of the coast of Jeju Island, Korea, this strain was cultured in several different media. When it was cultivated in static liquid conditions four new prenylated diphenyl ethers (**172**–**175**) were biosynthesized. This is the first report of the isolation of prenylated diphenyl ethers from a member of the genus *Aspergillus*. Biological tests indicated that compound **174** displayed moderate cytotoxicity in vitro against Hela and K562 cancer cell lines with IC_50_ values of 31.5 and 48.9 µM, respectively, whereas compound **175** exhibited moderate activity only against the Hela cell line [81]. 

The deep-sea-derived fungus *Cladosporium sphaerospermum* 2005-01-E3 was first cultured on a rice medium identifying the fungal hybrid polyketides cladosins A-E (**176**–**180**) [82]. This study was the first part of a project looking into structural diversity using the OSMAC approach. The HPLC-UV profile of the extract from the soybean-based medium indicated more cladosin derivatives, cladosins F (**181**) and G (**182**). These latter two compounds were evaluated for antiviral activity against the influenza A H1N1 virus, and for antitumoral, antitubercular and NF-kB inhibitory activities, but neither compound was found to be active [83].

Dong et al. [84] also employed the same strategy to explore the secondary metabolites of the sea mud-derived fungus *Ascotricha* sp. ZJ-M-5. Three new polyketides, ascotrichols A (**183**) and B (**184**), and ascotrichrone A (**185**), were isolated from the solid culture of the fungus on rice media. 

*Scopulariopsis* sp., a marine-derived fungus isolated from the Red Sea hard coral *Stylophora* sp., was subjected to OSMAC analysis to study its metabolic potential. In a previous study, several xanthones, bisabolene-type sesquiterpenoids, alkaloids and polyketides were isolated from solid rice cultures of the fungus [85]. Elnaggar et al. [86] identified a new naphthoquinone derivative (**186**) when they fermented the fungus on a solid white bean medium. It was evaluated for its cytotoxic, antitubercular and antimicrobial activity but did not show significant activity.

In their search for bioactive compounds from marine-derived fungi, Luan et al. altered culture parameters and conditions of the fungus *Spicaria elegans* KLA03 isolated from marine sediments collected in China. These authors found that the HPLC-UV profile of the extract changed dramatically when the strain was cultured on a modified mannitol-based medium (using NH_4_Cl as a nitrogen source). The aromatic polyketide eleganketal A (**187**) possessing a rare highly oxygenated ring system was isolated [87]. 

**Figure 7:**
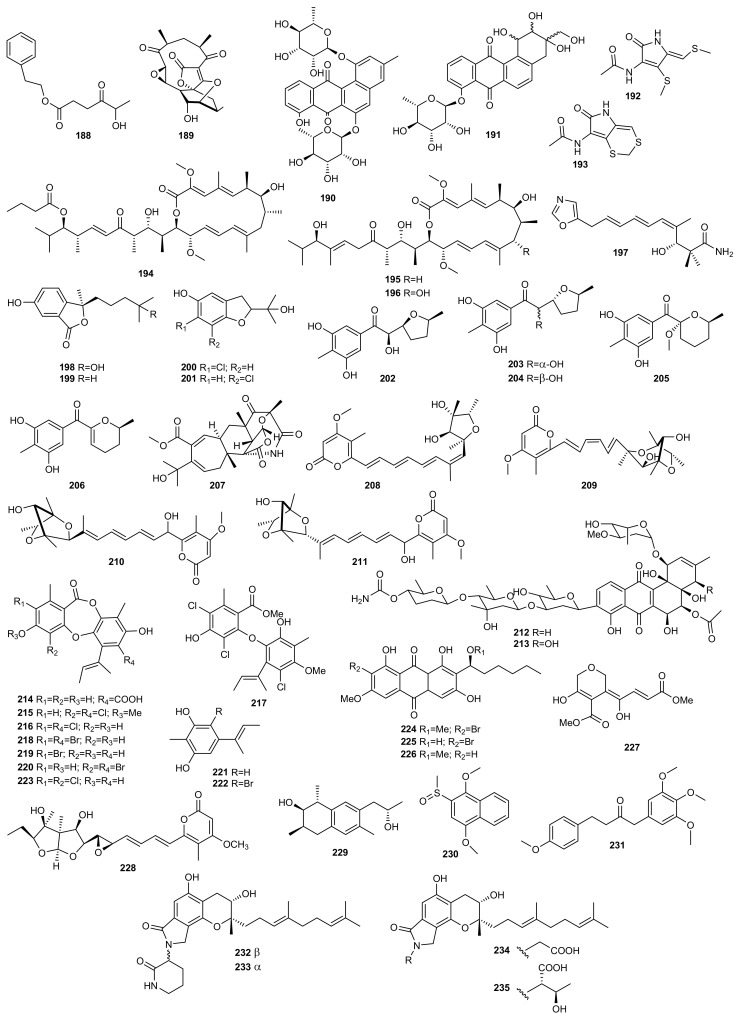
Cryptic polyketides (**188**–**235**) from marine-derived microorganisms obtained using OSMAC approach.

The marine sponge-derived fungus *Arthrinium arundinis* ZSDS1-F, collected from the Xisha Islands in China, was shown to metabolize the new natural product phenethyl 5-hydroxy-4-oxohexanoate (**188**) when the culture conditions were changed from rotary shaker to static fermentation on a rice medium [88] (Figure 7).

Marine actinomycetes have proven to be a rich source of novel secondary metabolites with various biological activities. Thus, based on the antimicrobial bioactivity exhibited by the metabolites isolated from *Verrucosispora*, a genus of rare actinomycetes, Zhang et al. [89] used the OSMAC approach to isolate new metabolites from the marine-derived strain *Verrucosispora* sp. MS100137. In this study, sixteen media were used to compare the metabolic profile of the bacterium. The extracts from each culture were analysed by HPLC and the fermentations whose extract showed the highest metabolic chemical diversity were scaled up and a new epoxidized polyketide, abyssomicin Y (**189**), was isolated. This compound exhibited a potent inhibitory effect against the influenza A virus (H1N1).

Abdelmohsen et al. [90] selected the best conditions to culture the sponge-associated bacterium *Actinokineospora* sp. EG49 in line with the OSMAC approach using statistical methods, high resolution Fourier transform mass spectrometry (HRFTMS), and nuclear magnetic resonance (NMR) spectroscopy. Four different fermentation approaches were used: ISP2 agar, ISP2 liquid broth, ISP2 liquid broth with Amberlite XAD-16, and ISP2 liquid broth with calcium alginate beads, as well as two different extraction procedures. The extracts of *Actinokineospora* sp. strain EG49 cultivated in the different culture media showed antitrypanosomal activity and therefore were analysed by HRFTMS using the Exactive-Orbitrap and high resolution NMR. This resulted in the isolation of two new *O*-glycosylated angucyclines named actinosporins A (**190**) and B (**191**). The active antitrypanosomal compound actinosporin A (**190**) was produced at the highest yield in ISP2 liquid broth and ISP2 liquid broth with calcium alginate helping with the dereplication.

The angucycline group of antibiotics belongs to a specific group of polycyclic aromatic polyketides derived from naturally occurring quinone saccharide antibiotics which exhibit mainly anticancer and antimicrobial activities. Both compounds (**190**, **191**) were tested against the parasites *Leishmania major*, *Trypanosoma brucei brucei,* and *Plasmodium falciparum,* and for cytotoxicity against J774.1 macrophages. Antitrypanosomal activity after 48 and 72 h was detected for actinosporin A (**190**), with no cytotoxicity against J774.1 macrophages. However, actinosporin B (**191**) was found to be inactive against *T. brucei*. 

Following the OSMAC approach, Ding et al. [91] isolated two new holomycin derivatives (**192**–**193**) from a marine-derived bacterium *Streptomyces* sp. DT-A37. Results revealed that there was a significant difference in secondary metabolite profile depending on whether the strain was cultured on a solid or liquid fermentation medium. The liquid medium yielded the new holomycin derivative **192**. Purification of the solid culture extracts led to one more new derivative of holomycin (**193**). 

The strain *Streptomyces* sp. HZP-2216E isolated from marine green algae *Ulva pertusa*, a traditional Chinese medicine, was cultured in three different media, 2216E solid medium [40], GMSS liquid medium [40], and GYM solid medium [41]. While the culture of this strain on GMSS liquid medium resulted in the isolation of the new 23-*O*-butyrylbafilomycin D (**194**), its culture on GYM solid medium led to the previously undescribed 21,22-en-bafilomycin D (**195**) and 21,22-en-9-hydroxybafilomycin D (**196**). Compounds **194**–**196** significantly suppressed the proliferation of several glioma cell lines and exhibited potent antibacterial activity against MRSA.

Wu et al. [13] used the OSMAC approach to stimulate secondary metabolite production of the new deep-sea sediment-derived *Streptomyces* sp. YB104 collected from the South Atlantic Ocean, leading to the discovery of a bioactive compound named inthomycin B (**197**). A total of seven organic extracts were obtained by a cultivation-dependent approach and changing media type. Analysis of their chemical composition identified their main components as inthomycins, reported to be highly specific inhibitors of cellulose biosynthesis. *Streptomyces* sp. YB104 is the first reported naturally occurring strain to produce a notably high yield of the new inthomycin B (**197**). 

The fungal strain *Pseudallescheria boydii*, isolated from the starfish *Acanthaster planci* collected in Hainan, was cultured on a glucose-peptone-yeast extract (GPY) medium, resulting in the isolation of two new isobenzofuranone derivatives, pseudaboydins A (**198**) and B (**199**) [92]. However, when the salinity of the medium was increased, the fungus produced two new benzofuran derivatives, 6-chloro-2-(2-hydroxypropan-2-yl)-2,3-dihydro-5-hydroxybenzofuran (**200**) and 7-chloro-2-(2-hydroxypropan-2-yl)-2,3-dihydro-5-hydroxybenzofuran (**201**) [93].

Pseudaboydin A (**198**) was moderately cytotoxic against human nasopharyngeal carcinoma cell lines HONE1 and SUNE1, and human glandular lung cancer cell line GLC82, with IC_50_ values of 37.1, 46.5, and 87.2 μM, respectively [92].

Among the cultivation-based approaches, thermo change is one of the more effective strategies in triggering silent biosynthetic expression systems to expand the number of fungi-derived natural products. Thus, Liu et al. isolated new polyketides named raistrickiones A-E (**202**–**206)** in response to lowering the fermentation temperature from 28 °C to 15 °C, with all other conditions remaining unchanged, from the strain JH-18 of *Penicillium raistrickii* isolated from marine saline soil of the coast of Bohai Bay in China. Compounds **202**–**206** exhibited moderate radical scavenging activity against DPPH [94].

While metals have traditionally been thought to be a problem for the synthesis of secondary metabolites from microorganisms, recent studies have shown that they can induce the production of interesting bioactive metabolites. Thus, Ding et al. applied the metal stress method to the marine-derived fungus strain *Aspergillus* sp. WU 243 collected from a hydrothermal vent in Taiwan. As a result, aspergstressin (**207**), a new polyketide-terpenoid hybrid, was isolated as a novel stress metabolite induced by a cobalt ion [30].

Auckloo et al. [95] successfully demonstrated the utility of the metal stress strategy for activating silent gene clusters and the subsequent isolation of unique NPs with potent antimicrobial properties. Thus, the marine fungus *Penicillium* sp. BB1122 from the Zhoushan Coast, China, was cultured applying specific concentrations of particularly heavy metals (cobalt, manganese, chromium, nickel, zinc, and cadmium) acting as elicitors and inducing the production of novel secondary metabolites. The optimal stress conditions were determined as 6 mM cobalt ion concentration based on the HPLC profile of the extract, leading to the isolation of four novel polyketide compounds, neocitreoviridin (**208**), 10*Z*-isocitreoviridinol (**209**), penicillstressol (**210**) and isopenicillstressol (**211**). These compounds had potent antibiotic properties against MRSA and *Pseudomonas aeruginosa*. 

In a quest to discover new secondary metabolites, Akhter et al. [74] successfully used the same strategy to stimulate the cryptic gene cluster of marine microorganisms. Different metal ions were applied to the marine strain *Streptomyces pratensis* NA-ZhouS1 isolated from marine sediment in the waters along the Zhoushan Coast in East China. One of these, 100 µM nickel ion (NiCl_2_⋅6H_2_O), led to the isolation of two new aromatic polyketides, stremycin A (**212**) and B (**213**). These novel antibiotics exhibited antimicrobial activity against *Pseudomonas aeruginosa*, MRSA, *Klebsiella pneumoniae*, *Bacillus subtilis* and *Escherichia coli*. 

When microorganisms are exposed to different types of media supplemented with halogens, they can activate their biosynthetic pathways to restore osmotic imbalance, thus activating different sets of hidden genes. The marine-derived fungus *Aspergillus unguis* CRI282-03, isolated from a sponge collected in Thailand, produced three new chlorinated depsidones (**214**–**216**) and a new diaryl ether (**217**) when it was cultured in PDB medium prepared with seawater [28]. However, this fungal strain produced three new brominated depsidones (**218**–**220**) and two new orcinol derivatives (**221**,**222**) when cultured on KBr medium, and a new depsidone (**223**) on KI broth. Bioassay results indicated that compounds **218**–**220** inhibited aromatase, a therapeutic target for the treatment of breast cancer [96].

Similarly, to determine whether the biosynthetic machinery of the fungus *Aspergillus* sp. SCSIO F063, isolated from a marine sediment sample collected in the South China Sea, had the potential to produce halogenated compounds, the fungus was fermented in PDB supplemented with 3% NaBr. As expected, two new brominated anthraquinones (**224**, **225**) were obtained from the methanolic extracts, together with a new nonhalogenated anthraquinone (**226**). New compounds were evaluated for their cytotoxic activity against human breast adenocarcinoma MCF-7, human glioblastoma SF-268, and human large-cell lung carcinoma NCI-H460 cell lines. The metabolite 6-*O*-methyl-7-chloroaveratin (**226**) displayed the strongest inhibition activity. These compounds were also tested for their antibacterial activity against three Gram positive bacteria, *Staphylococcus aureus* ATCC 29213, *Bacillus thuringiensis* ATCC 39765, and *Bacillus subtilis* ATCC 6633, but none of them exhibited high inhibition rates [97]. 

The new polyketide isariketide (**227**) was obtained from the fungus *Isaria felina* (current name: *Beauveria felina)* KMM 4639, isolated from a marine sediment sample collected in the South China Sea when it was cultivated with a high concentration of KBr. That compound’s effect on viability and apoptosis induction of human prostate cancer cells and murine non-malignant splenocytes and erythrocytes was studied but did not exhibit cytotoxic activity at concentrations of up to 100 μM [98]. 

To obtain new metabolites, the gorgonian-derived fungus *Aspergillus* sp. SCSGAF 0076 (re-numbered as SCSIO 41501) was cultivated in a basic culture medium supplemented with different concentrations of l-tryptophan. The addition of 0.05% of l-tryptophan led to a new asteltoxin B (**228**), which was absent in the medium supplemented with 0.2% of the amino acid. This compound was tested for antibacterial activity (*Bacillus subtilis* and *Escherichia coli*) and enzyme-inhibitory activity (cathepsin B enzymes, human monoacylglycerol lipase, human leukocyte elastase, inosine monophosphate dehydrogenase, proteasome, protein tyrosine phosphatase 1B, and Src homology 2 domain containing phosphotyrosinephosphatase 2), but no positive results were found [72].

Following the same strategy, the marine derived fungus *Dichotomomyces cejpii* (current name: *Aspergillus cejpii*) F31-1 was cultured in GPY medium supplemented with amino acids. The new polyketides dichocetides A-C (**229**–**231**) were isolated by adding l-tryptophan and l-phenylalanine [57,58].

Based on the biosynthetic process of isoindolinones, Yin et al. [99] developed a rational supply strategy of amino compounds for the production of novel derivatives from marine fungus *Stachybotrys longispora* FG216. Isoindolinones are formed by a spontaneous reaction between a phthalic aldehyde precursor and an ammonium ion or amino compounds. Ammonium ions can be replaced by different amino compounds to create structural diversity in the biosynthetic process of isoindolinones. On that basis, they tested four amino compound precursors, (±)-3-amino-2-piperidinone, (3*S*)-3-amino-2-piperidinone hydrochloride, glycine, and l-threonine, resulting in four new isoindolinone derivatives, FGFC4-FGFC7 (**232**–**235**). Enantiomers **232** and **233** were the products of (3*S*)-3-amino-2-piperidinone and (3*R*)-3-amino-2-piperidinone by comparing them with the optical product derived by feeding of (3*S*)-3-amino-2-piperidinone hydrochloride. Compounds **234** and **235** were derived from the feeding of glycine and l-threonine, respectively. Among the four derivatives, FGFC6 (**234**) and FGFC7 (**235**) showed promising fibrinolytic activities. 

### 4.2. Epigenetic Approach

**Figure 8:**
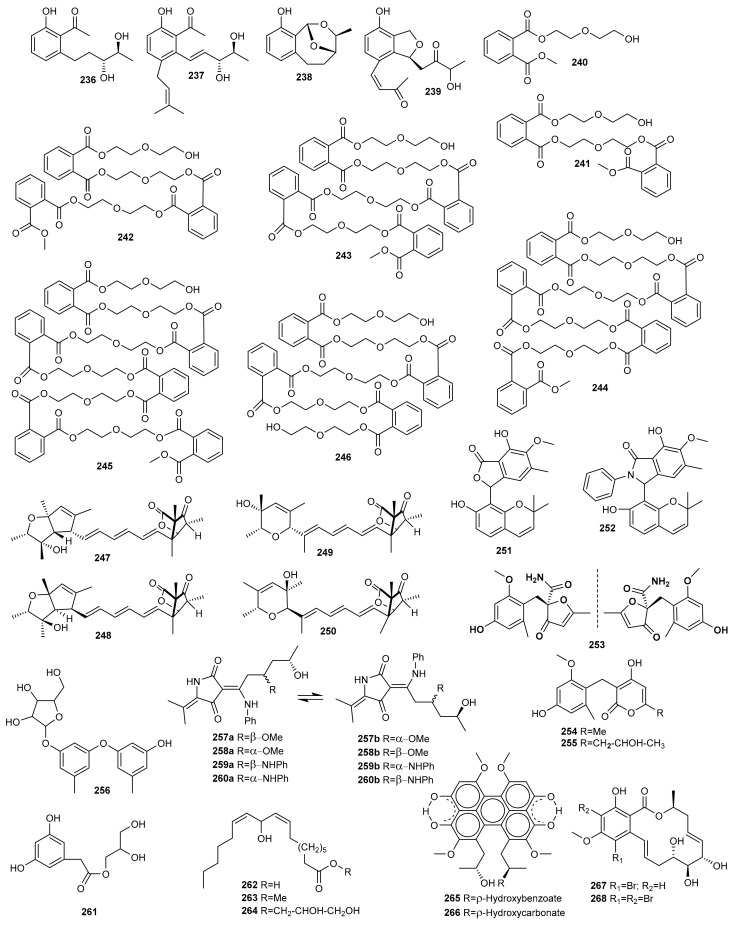
Cryptic polyketides (**236**–**268**) from marine-derived microorganisms obtained using the epigenetic approach.

The DNMT inhibitors 5-aza-2-deoxycytidine and RG 108 were used to obtain four new polyketides from the marine-derived fungus *Pestalotiopsis* sp., named pestalotiopols A–D (**236**–**239**) (Figure 8). The new compounds were evaluated for their cytotoxic activity against four human carcinoma cell lines, a human gastric carcinoma cell line (BGC-823), a human hepatocellular carcinoma cell line (SMMC-7721), a human carcinoma cell line (Ichikawa), and a human kidney cancer cell line (7860). Compounds **236** and **237** showed best results with IC_50_ values of 16.5–56.5 mM [100].

There are other reports of the addition of a DNMT inhibitor (5-AZA) as an epigenetic modifier in culture medium leading to the production of new metabolites. Examples include novel diethylene glycol phthalate esters (**240**–**246**) from the marine-derived strain *Cochliobolus lunatus* (current name: *Curvularia lunata*) TA26-46, isolated from tissue from the inner part of the sea anemone *Palythoa haddoni*, collected from the Weizhou coral reef in the South China Sea. Compounds **241**–**246** are the first examples of naturally occurring phthalate ester oligomers [101].

Along these same lines, He et al. isolated four new polyketides, varilactones A (**247**) and B (**248**), and wortmannilactones M (**249**) and N (**250**), from the fungus *Penicillium variabile* HXQ-H-1, isolated from mangrove rhizosphere soil collected along the coast of Fujian Province (China), when it was cultivated on potato-based medium with the HDAC inhibitor SAHA. Varilactones A (**247**) and B (**248**) have a novel skeleton with a triene unit linking an oxabicyclo[2.2.1] heptane and an oxabicyclo[3.3.0] octane ring. Cytotoxicity against Hela, MGC-803 cell lines and antiviral activity against influenza A virus (H1N1) were evaluated, although none of the compounds showed activity [102].

The marine-derived fungus *Asteromyces cruciatus* was subjected to both epigenetic modification and osmotic stress to enhance the production of new natural products [103]. *A. cruciatus* was fermented under twelve treatment conditions, adding epigenetic modifiers, compounds for abiotic stress, and the two in combination. These strategies led to the isolation and characterization of two new polyketides from fermentation with SAHA and high concentrations of NaCl, primarolides A (**251**) and B (**252**). These new polyketides were tested for antimicrobial activity against human pathogen microorganisms such as *Staphylococcus aureus* (MRSA, ATCC 33591), *S. warneri* (ATCC 17917), *Enterococcus faecium* (VRE, EF379), *Pseudomonas aeruginosa* (ATCC 14210), *Proteus vulgaris* (ATCC 12454) and *Candida albicans* (ATCC 14035) but with no positive results.

An epigenetic modification of a culture of the marine-derived fungus *Aspergillus terreus* RA2905 using the HDAC inhibitor SAHA, resulted in the isolation of a pair of new enantiomers, (+)- and (−)-asperfuranone (**253**), together with two new benzyl pyrones, asperpyranones A (**254**) and B (**255**). All compounds isolated were tested for their antifungal, antibacterial, cytotoxic, and PTP1B inhibitory activities. Compounds (±)-**253**, (+)-**253**, (−)-**253**, and **254** exhibited antifungal activity against *Candida albicans* with MIC values between 16 and 64 µg/mL, and PTP1B inhibitory activity with IC_50_ values of 17.32–46 µM. Compound **254** also exhibited good antibacterial activity against *Pseudomonas aeruginosa*. None of the isolated compounds showed significant cytotoxic activity [104].

Another new diphenylether-*O*-glycoside (**256**) was produced by the marine-derived fungus *Aspergillus* sp. SCSIOW3, isolated from a deep marine sediment sample collected in the South China Sea, when this strain was exposed to chemical epigenetic manipulation with the integration of HDAC inhibitor (suberohydroxamic acid) and DNMT inhibitor (5-AZA). This compound was the first diphenyl ether glycoside incorporating d-ribose as a sugar component. Bioactivity tests indicated that the glycosylated compound **256** exhibited protective activity towards free radicals [105].

Subsequently, new tetramic acids were obtained by Zhang et al. [106] by adding the epigenetic modifier SAHA to the cultures of the sediment-derived fungus *Cladosporium sphaerospermum* L3P3. These researchers showed how the addition of an epigenetic modifier activated gene clusters to generate four new tetramic acids, cladosins H−K (**257**–**260**) containing aniline moieties. Interestingly, all of the compounds **257**–**260** existed as two tautomeric forms (**a** and **b**) differing in the configurations of the enamine. Their cytotoxicity was evaluated against the PC-3, MGC-803, SH-SY5Y, HCT-116, K562, and HL-60 cell lines. Cladosin I (**258**) exhibited promising cytotoxicity against the HL-60 cell line.

The marine-derived fungus *Leucostoma persoonii* (current name: *Cytospora leucostoma*) isolated from the red mangrove *Rhizophora mangle* obtained from the Florida Everglades, was cultivated on a large scale with epigenetic modifiers. Extracts of this strain exhibited important antimalarial and antibiotic activity. Using sodium butyrate as an HDAC inhibitor resulted in the increased yield of known cytosporones and the production of a previously undescribed cytosporone R (**261**) [107].

An epigenetic approach was chosen to explore the cryptic metabolites of the fungus *Cladosporium cladosporioides*, isolated from a tidal pool along the coast of Casco Bay in Portland. Treatment of this fungal strain with 5-AZA elicited the *de novo* production of several oxylipins, (9*Z*,12*Z*)-11-hydroxyoctadeca-9,12-dienoic acid (**262**), its methyl ester (**263**), and glycerol conjugate (**264**). In contrast, SAHA induced the production of two perylenequinones, cladochromes F (**265**) and G (**266**) [108].

This same strategy was applied to the marine-derived fungus *Cochliobolus lunatus* (Current name: *Curvularia lunata*) TA26-46, isolated from the sea anemone *Palythoa haddoni*. Culture of this fungal strain in starch medium treated with 0.01 M sodium butyrate, an HDAC inhibitor, resulted in the isolation of two new 14-membered resorcylic acid lactones, 5-bromozeaenol (**267**) and 3,5-dibromozeaenol (**268**). These new compounds were evaluated for their cytotoxicity, antifouling activity and zebrafish embryo teratogenicity. Unfortunately, none of them showed activity in these bioassays [109].

## 5. Terpenes

### 5.1. OSMAC Approach

**Figure 9:**
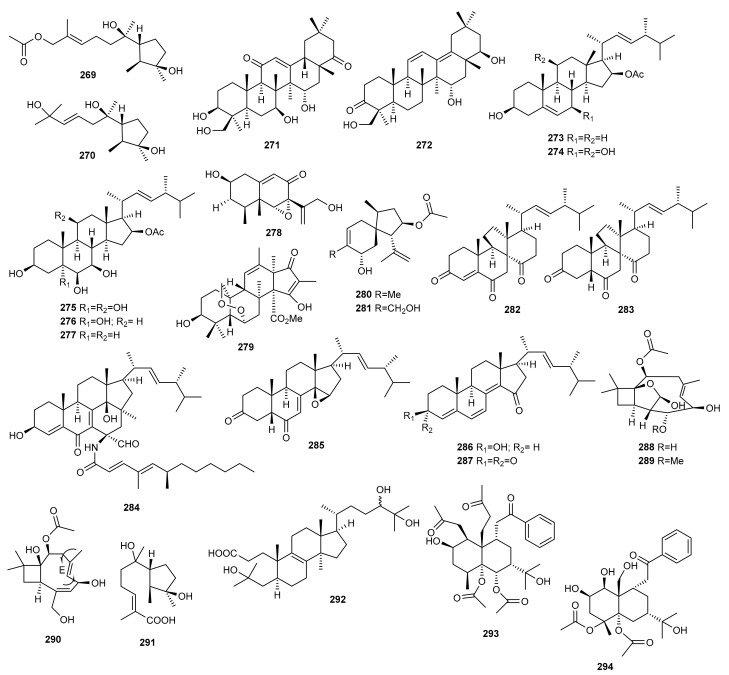
Cryptic terpenes (**269**–**294**) from marine-derived microorganisms obtained using the OSMAC approach.

In marine environments, terpenes display an array of diverse chemical structures with promising biological activities. To discover new metabolites, the marine-derived fungus *Trichothecium roseum*, isolated from marine driftwood from the intertidal zone of Lingshan in China, was cultured in two different media [68]. Culture on rice medium was assayed, leading to the isolation of two new cyclonerodiol sesquiterpenes (**269**, **270**) (Figure 9). These compounds were evaluated for cytotoxic and nematicidal activity and brine shrimp (*Artemia salina*) toxicity, as well as antifungal activity, but did not exhibit noteworthy activity.

Elnaggar et al. [86] identified two new triterpenoids (**271**,**272**) during the OSMAC analysis of the marine-derived fungus *Scopulariopsis* sp. fermented on a solid white bean medium. None of the metabolites isolated in this study was detected when the fungus was cultivated on rice medium, thereby proving the power of the OSMAC approach as applied in this study. Both compounds (**271**,**272**) were evaluated for their cytotoxic, antitubercular and antimicrobial activities, but none of them showed significant activity.

New penicisteroids D-H (**273**–**277**) were isolated from the deep-sea-derived fungus *Penicillium granulatum* MCCC 3A00475 following the same strategy. Different culture conditions were studied and secondary fungal metabolism changed dramatically under shaken fermentation conditions in liquid medium [32]. These compounds could have an important function as analogues to Retinoid X receptor-α (RXR*a*) transcriptional inhibitors, that play an important role in the treatment of cancer and metabolic and neurodegenerative diseases. The crude extract showed potent cytotoxicity against several cancer cells. Compounds **274**, **276** and **277** showed moderate selective inhibitory effects against 12 different cancer cell lines with IC_50_ values of around 5 µM. The study of the mechanism suggested they were not only capable of inducing apoptosis through an RXRα-dependent pathway but also inhibited proliferation by cell cycle arrest at the G0/G1 phase.

The new compounds isophomenone (**278**) and 3-deacetylcitreohybridonol (**279**) were detected when the marine-derived fungus *Penicillium commune* QSD-17, isolated from a sediment sample collected in the South China Sea, was grown on solid rice medium under static conditions. These compounds were absent when this fungal strain was cultured in PDB medium. [110].

Chemical analysis of the marine sponge-derived fungus *Penicillium adametzioides* AS-53, collected on Hainan Island in the South China Sea, led to the isolation of two new acorane sesquiterpenes, adametacorenol A (**280**) and B (**281**), when it was cultured on static rice medium. In contrast, none of these metabolites were detected when the fungus was cultivated in a liquid PDB. The absolute configuration of compound **280** was determined by the modified Mosher’s method [26].

The *Halichondria* sponge-derived fungus *Gymnascella dankaliensis* (current name: *Arachniotus dankaliensis*) was cultured in two different culture media. A modified malt extract medium containing soluble starch instead of glucose resulted in two extremely unusual steroids, dankasterones A (**282**) and B (**283**). In contrast, four additional unusual steroids, gymnasterones A-D (**284**–**287**), were isolated from the original malt extract medium. The steroids **282**, **283**, and **285**–**287** exhibited significant and marginal growth inhibition against the murine P388 cell line with ED_50_ values of 2.2, 2.8, 1.6, 0.9, and 2.5 µg/mL, respectively. In addition, compound **282** showed appreciable growth inhibition against human cancer cell lines (MG-MID–5.41) [22].

The marine-derived fungus *Ascotricha* sp. ZJ-M-5, isolated from a mud sample collected on a coastal beach in China, was subjected to OSMAC analysis. It was cultured along with Mg^2+^, leading to the isolation of three new caryophyllene derivatives (**288**–**290**) [111]. Their cytotoxic activity was tested against human leukemia HL-60 and K562 cells showing more potent activity than the positive control cisplatin. In contract, when *Ascotricha* sp. ZJ-M-5 was cultivated in a eutrophic medium using sea salt, a new cyclonerol derivative (**291**) [112] and a new 3,4-seco-lanostane triterpene (**292**) [113] were detected.

Two new sesquiterpenes, 1β,5α,6α,14-tetraacetoxy-9α-benzoyloxy-7β*H*-eudesman-2β,11-diol (**293**) and 4α,5α-diacetoxy-9α-benzoyloxy-7β*H*-eudesman-1β,2β,11,14-tetraol (**294**), were produced as stress metabolites in the cultured mycelia of *Pestalotiopsis* sp. Z233, isolated from the algae *Sargassum horneri*, in response to abiotic stress elicitation by CuCl_2_. New compounds **293** and **294** showed tyrosinase inhibitory activities with IC_50_ values of 14.8 μM and 22.3 μM, respectively. Tyrosinase inhibitors can be clinically useful for the treatment of some dermatological disorders associated with melanin hyperpigmentation [114].

### 5.2. Epigenetic Approach

**Figure 10:**
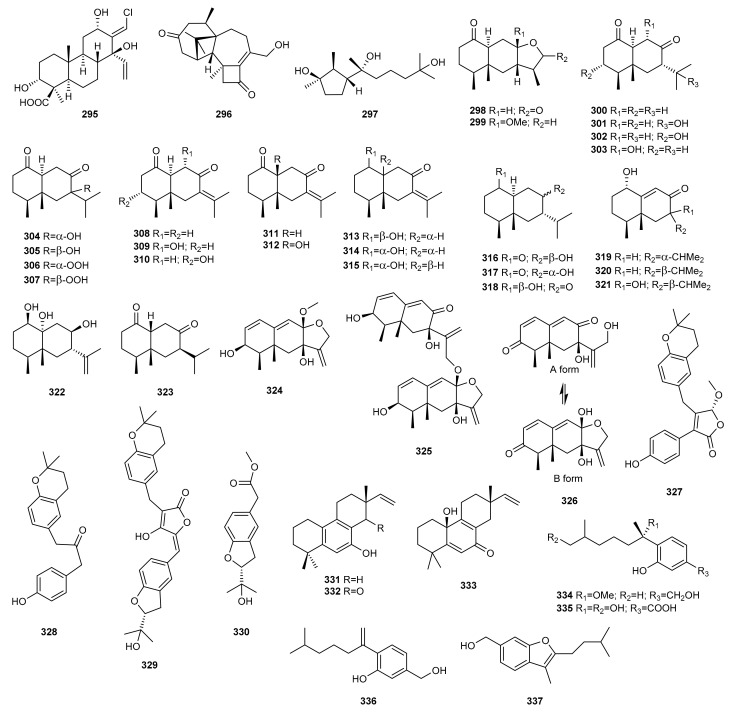
Cryptic terpenes (**295**–**337**) from marine-derived microorganisms obtained using epigenetic approach.

Among the different methods used to regulate the production of secondary metabolites from marine fungi, chemical epigenetic manipulation has proven to be a promising strategy to wake the silent biosynthetic gene clusters and obtain novel compounds. Thus, the marine-derived fungus *Trichoderma harzianum* (XS-20090075), isolated from a soft coral collected from the South China Sea, was subjected to chemical epigenetic manipulation with sodium butyrate [115]. The new compounds, the cleistanthane diterpenoid harzianolic acid A (**295**), the harziane diterpenoid harzianone E (**296**), and the cyclonerane sesquiterpenoid, 3,7,11-trihydroxy-cycloneran (**297**), were evaluated as antimicrobial agents against bacterial strains (*Staphylococcus aureus* ATCC 27154, *Escherichia coli* ATCC 25922, *Pseudomonas aeruginosa* ATCC 10145, *P. fulva* ATCC 31418, *Aeromonas salmonicida* ATCC 7965D, *Vibrio harveyi* ATCC BAA-2752, *Photobacterium angustum* ATCC33975, *Enterobacter cloacae* ATCC 39978, and *E. hormaechei* ATCC 700323), and one pathogenic fungal strain (*Candida albicans* ATCC 76485). Only **296** exhibited weak antibacterial activity against *P. angustum*. This was the first time that a chlorinated cleistanthane diterpenoid was obtained from the genus *Trichoderma* (Figure 10).

Niu et al. [116] used chemical epigenetic manipulation and the HDAC inhibitor SBHA to isolate 26 new eremophilanes, namely eutyperemophilanes A-Z (**298**–**323**), from the deep-sea-derived fungus *Eutypella* sp. MCCC 3A00281 obtained from sediment in the Southern Atlantic Ocean. All compounds were evaluated for their inhibitory effect on nitric oxide production, and the potent activity of compounds **306** and **307** suggests they should be further evaluated as anti-inflammatory agents.

Taking into account the positive results obtained with epigenetic manipulation to isolate new metabolites in other marine-derived fungi, Wang et al. [117] fermented the deep-sea marine-derived fungus *Aspergillus* sp. SCSIOW2, treated with a combination of HDAC inhibitor (suberohydroxamic acid) and DNMT inhibitor (5-AZA). This resulted in the isolation of three new eremophilane-type sesquiterpenes, dihydrobipolaroxin B (**324**), dihydrobipolaroxin C (**325**) and dihydrobipolaroxin D (**326**). These compounds were not produced in the untreated cultures, but **324** and **325** could be artificial. All three compounds exhibited moderate nitric oxide inhibitory activity but had no cytotoxic effects.

In order to enrich the chemodiversity of secondary metabolites the marine algicolous fungus *Aspergillus terreus* OUCMDZ-2739 was fermented in the presence of trichostatin A (TSA), an HDAC inhibitor. This resulted in the isolation of four new meroterpenoids identified as (*R*)-4-((2,2-dimethylchroman-6-yl)methyl)-3-(4-hydroxyphenyl)-5-methoxyfuran-2(5H)-one (**327**) [118], 1-(2,2-dimethylchroman-6-yl)-3-(4-hydroxyphenyl)propan-2-one (**328**), (*R*,*E*)-3-(2,2-dimethylchroman-6-yl)-4-hydroxy-5-((2-(2-hydroxypropan-2-yl)-2,3-dihydrobenzofuran-5-yl)methylene)furan-2(5H)-one (**329**), and methyl (*R*)-2-(2-(2-hydroxypropan-2-yl)-2,3-dihydrobenzofuran-5-yl) acetate (**330**) [118]. Of the new compounds, only compound **329** exhibited stronger α-glucosidase inhibition than the positive controls, but none exhibited cytotoxic activity.

In another study, the algicolous fungus *Aspergillus wentii* na-3, isolated from the tissue of the brown alga *Sargassum fusiforme*, was fermented along with SAHA. Using an HPLC-monitored separation, three new norditerpenes, aspewentins A−C (**331**−**333**), were identified from the extract [119].

Aspewentins A−C (**331**−**333**) were assayed for growth inhibition against one species of marine zooplankton (*Artemia salina*) and three marine phytoplankton species (*Chattonella marina*, *Heterosigma akashiwo*, and *Alexandrium* sp.), all exhibiting potent bioactivity. Compound **333** was more active against *C. marina* and *H. akashiwo*, **332** was most toxic to *A. salina*, and **333** was more active against *Alexandrium* sp.

Another fungus from the *Aspergillus* genera, *Aspergillus sydowii*, obtained from a marine sediment in Taiwan, was also cultivated adding a DNMT inhibitor, 5-AZA, to its fermentation culture. Three new bisabolane-type sesquiterpenoids, (7*S*)-(+)-7-*O*-methylsydonol (**334**), (7*S*,11*S*)-(+)-12-hydroxysydonic acid (**335**) and 7-deoxy-7,14-didehydrosydonol (**336**), were isolated [120]. These compounds were evaluated for their anti-diabetic and anti-inflammatory activities. Of these new isolates, compounds **334** and **335** were the most promising.

The marine-derived fungus *Aspergillus versicolor* XS-20090066, isolated from the gorgonian *Dichotella gemmacea* collected in the South China Sea, was cultivated in Czapek-Dox liquid medium using a combination of the HDAC and DNMT inhibitors SAHA and 5-AZA, respectively. A new bisabolane sesquiterpene, aspergillusene E (**337**), was isolated from the extracts and identified [121]. Compound **337** exhibited antibacterial activity against *Staphylococcus epidermidis* and *S. aureus* and antifungal activity against *Candida albicans* and *C. tropicalis*. The antifouling assay was performed using bryozoan larvae of *Bugula neritina* and compound **337** exhibited anti-larval activity [121].

## 6. Miscellaneous

### 6.1. OSMAC Approach

**Figure 11:**
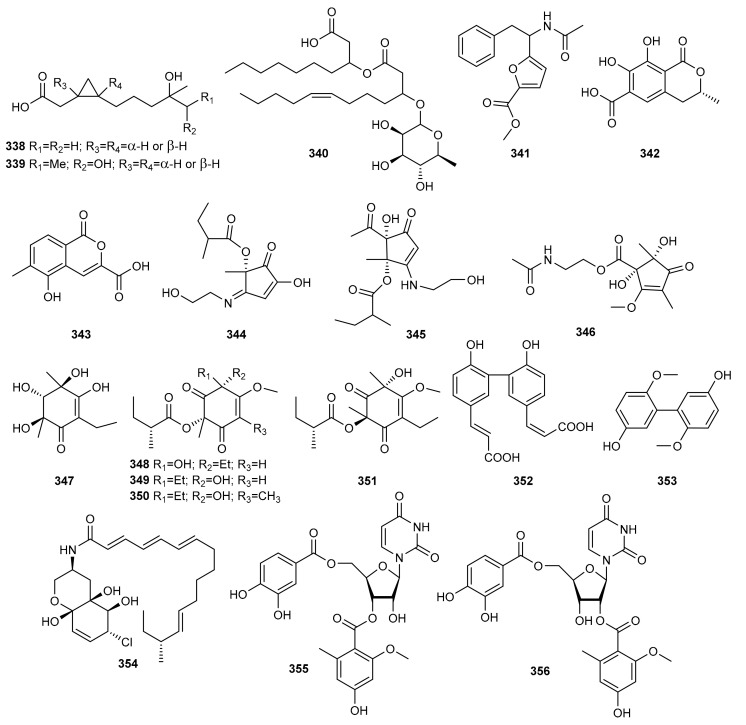
Cryptic metabolites (**338**–**356**) from marine-derived microorganisms obtained using the OSMAC and epigenetic approaches.

The OSMAC approach was used to produce two new cyclopropaneacetic acid derivatives (**338**, **339**) by the marine-derived bacterium, *Streptomyces* sp. DT-A37, isolated from marine sediments collected from Dongtou in China. There was a significant difference in secondary metabolite profile when the strain was cultured on a solid rice medium compared to a liquid GMSS medium. Purification of the solid culture extract led to the new cyclopropaneacetic acid derivatives **338** and **339** (Figure 11). These compounds were evaluated for their antimicrobial (*Escherichia coli* ATCC 25922, *Staphylococcus aureus* ATCC 25923, *Pseudomonas aeruginosa* ATCC 27853 and *Candida albicans* ATCC 10231), cytotoxic (H1975 cells), and inhibitory activities against BRD4 protein, but none exhibited any activity [91].

Similarly, the arctic marine *Pseudomonas* sp. strain M10B774 was cultured in four different liquid media in an attempt to activate biosynthetic pathways leading to the production of antibacterial and anticancer compounds, the result being a new mono-rhamnolipid (**340**) isolated from this strain. HR-ESI-MS analyses of the culture extracts showed that the isolated rhamnolipid was present in the samples from the M19, VR_1 and VR_2 rich media, but not in the SGC low nutrient medium [122].

Compound **340** showed high activity against *Enterococcus faecalis* only at the highest tested concentration of 150 µM. It also inhibited biofilm formation of *Staphylococcus epidermidis* and did not exhibit any activity against the human melanoma cancer cell line A2058 [122].

As part of a research effort to find bioactive compounds from marine-derived fungi, Uchoa et al. [123] reported on the chemical study of *Aspergillus niger* BRF-074, isolated from sediments from the Northeast coast of Brazil. Following the OSMAC strategy, they grew the fungal strain in three different culture media. A new ester furan derivative (**341**) was obtained from the MPDB medium, which failed to appear in PDB or PDYB media. This compound was cytotoxic against the HCT-116 cancer cell line with an IC_50_ value of 2.9 μM [123].

Similarly, Qi et al. [59,124] altered the medium composition of the marine gorgonian-derived fungus *Aspergillus* sp. SCSIO 41501 (formerly SCSGAF 0076) to activate silent biosynthetic genes leading to the biosynthesis of new compounds. Thus, an increase in the concentration of l-tryptophan from 0.05% to 0.2% in a basic culture medium led to the isolation of two new isocoumarins, aspergillspins F-G (**342**, **343**) [59]. Furthermore, fermentation of SCSIO 41501 strain on a solid rice medium instead of the liquid basic medium used previously, led to three new cyclopentenone derivatives, aspergispones A-C (**344**–**346**), and five new cyclohexenone derivatives, aspergispones D-H (**347**–**351**) [124].

These new compounds (**342**–**351**) were evaluated for their antibacterial activity against several human pathogens. Compounds **342** and **343** were also assayed for their cytotoxicity against human carcinoma cell lines and compounds **344**–**351** for their inhibitory activity against acetylcholinesterase and toxicity against brine shrimp. All the compounds tested in these assays proved to be inactive [59,124].

In the search for new structurally bioactive compounds from halotolerant fungi in hypersaline media, the effect of high salt stress on the marine-derived fungus *Spicaria elegans* KLA-03, isolated from marine sediments collected in Jiaozhou Bay in China, was studied. This strain was cultivated under conditions of 10% salinity, yielding the new metabolite (2*E*,2′*Z*)-3,3′-(6,6′-dihydroxybiphenyl-3,3′-diyl)diacrylic acid (**352**), which was not produced by *S*. *elegans* KLA-03 when cultivated in a low-salt (3%) medium. This compound showed moderate antibacterial activity against *Pseudomonas aeruginosa* and *Escherichia coli* with MIC values of 0.038 and 0.767 mM, respectively [125].

### 6.2. Epigenetic Approach

Zhu et al. [126] identified a new biphenyl derivative by epigenetic manipulation of the marine-derived fungus *Aspergillus versicolor* MCCC 3A00080, isolated from deep-sea Pacific Ocean sediment. Cultivation of this strain in PDB with SAHA, an HDAC inhibitor, led to versiperol A (**353**), which modestly inhibited the growth of *Staphylococcus aureus* with an MIC value of 8 μg/mL.

To tap the potential of marine-derived fungi to produce diverse secondary metabolites, He et al. tested several HDAC and DNMT inhibitors on some fungal strains. Among them, the *Penicillium variabile* (current name: *Talaromyces variabilis*) strain HXQ-H-1, isolated from mangrove rhizosphere soil collected on the coast of Fujian Province (China), was found to be sensitive to 5-AZA, causing new peaks in the HPLC-UV profile. Thus, a new and highly modified fatty acid amide, varitatin A (**354**), was isolated from this fungus. Its absolute configuration was established by Mosher’s method. Compound **354** was cytotoxic against HCT-116 cells with an IC_50_ value of 2.8 μM and also inhibited the effects of protein tyrosine kinases [127].

Wu et al. [120] also used a chemical epigenetic approach to trigger the chemical diversity of the marine-derived fungus *Aspergillus versicolor* XS-20090066 isolated from gorgonian *Dichotella gemmacea* collected from the Xisha Islands coral reef in China. This strain was cultivated in Czapek-Dox liquid medium using a combination of SAHA and 5-AZA. The HPLC profile of the fungal culture extract was significantly changed compared to the control with no epigenetic agent. Chemical epigenetic manipulation produced two new nucleoside derivatives, kipukasins K (**355**) and L (**356**). Compound **355** exhibited antibacterial activity against *Staphylococcus epidermidis* and *S. aureus* with MIC values of 8–16 µg/mL.

## 7. Conclusions

This review is an overview of all new cryptic metabolites from marine-derived microorganisms obtained by means of the principal cultivation-dependent approaches and other additional strategies available to produce these previously unexplored NPs. To date, more than 350 new cryptic compounds from marine microorganisms have been identified, especially polyketides and alkaloids.

The most widely used strategy has been the OSMAC approach, possibly because it is simple, quick and effective in enhancing the chemo-diversity of marine NPs by activating silent gene clusters. However, given the high number of culture modifiable conditions, this technique leads to large sets of fractions difficult to analyze by conventional methods. Nowadays, some authors are working to improve the OSMAC approach by combination with other techniques, such as Log P and NMR fingerprinting [128].

So far, only around 25% of the new cryptic metabolites from marine microorganisms have been isolated using enzyme inhibitors, chemical elicitors and epigenetic modifiers. These techniques are an interesting resource and need to be studied further, as they could be developed into a new and more powerful tool for the discovery of new NPs.
